# Correlates of adherence to exclusive breastfeeding while on anti-retroviral therapy among mothers living with HIV in Port Harcourt, Nigeria

**DOI:** 10.11604/pamj.2025.50.90.38845

**Published:** 2025-04-01

**Authors:** Egbe Bassey Etowa, Felix Emeka Anyiam, Glory Osandatuwa, Batholomew Chibuike James, Josephine Etowa

**Affiliations:** 1 Ontario HIV Treatment Network, Toronto, Ontario, Canada,; 2 Daphne Cockwell School of Nursing, Faculty of Community Services, Toronto Metropolitan University, Toronto, Ontario, Canada,; 3 Centre for Health and Development, College of Health Sciences, University of Port Harcourt, Port Harcourt, Nigeria,; 4 Durban University of Technology, Durban, South Africa,; 5 School of Nursing, Faculty of Health Sciences, University of Ottawa, Ottawa, Canada,; 6 Sheffield Hallam University, Public Health Program, Sheffield, United Kingdom

**Keywords:** Antiretroviral therapy, exclusive breastfeeding guideline, African mother, social support

## Abstract

**Introduction:**

in low- and middle-income nations like Nigeria, vertical transmission of HIV is still common. Although there are recommended guidelines for infant feeding for women living with HIV, the level of adherence has significantly varied across African women. This study assessed the adherence to exclusive breastfeeding (EBF) guidelines and its associated factors among nursing mothers living with HIV/AIDS in Port Harcourt, Nigeria.

**Methods:**

a descriptive cross-sectional study was carried out between March and August 2022 among nursing mothers living with HIV/AIDS. Structured and validated questionnaires were used to collect data from 400 participants and were analyzed for this purpose. Chi-square statistics; bivariate and multivariate logistic regression analyses were carried out at alpha 0.05 to determine the correlates of adherence to the national guideline of exclusive breastfeeding while on anti-retroviral therapy.

**Results:**

the majority of the respondents were within the age range 30-39 years, 66.0% with a mean age of 34.6±5.6. Most respondents know (90.7%) of the mothers knew about the EBF policy and 65% adhered to the policy guidelines. Being employed/self-employed (OR=2.22, p=0.001); knowledge of the guidelines (OR=6.3, p=0.001); and support from household (OR=2.39, p=0.003), father/spouse (OR=65.6, p=0.001), close relatives (OR=3.5, p=0.001), healthcare (OR=38.2, p=0.01) were all associated with adherence to EBF. After adjusting for confounders, using the multivariate logistic regression, only support from father/spouse (OR=23.24, p=0.001) and healthcare (OR=47.6, p=0.01) were strong predictors of adherence to EBF guidelines.

**Conclusion:**

inclusive education involving mothers, social support networks, and healthcare providers will increase adherence to national guidelines EBF among mothers living with HIV.

## Introduction

With 20 million women living with the virus and over 2 million pregnancies among women who are HIV-positive each year, the HIV/AIDS epidemic is one of the main issues affecting women's health [1]. HIV infection has thus grown to be a serious issue that makes managing pregnancy more difficult. The prevalence of HIV varies greatly throughout Africa; in certain sub-Saharan African nations, about one in five expectant mothers is infected [2]. The national prevalence of HIV among pregnant women in Nigeria is reported to be 4.1%, with prevalence rates ranging from 3.0-7.5% in different parts of the country [3]. As of 2019, the prevalence in Nigeria was 1.4%, and it was estimated that 1.9 million Nigerians live with HIV [4]. In Zambia, it was estimated that there were 26,000 new HIV infections among young women (15- 49 years) compared to 19000 among young men in 2019 [5]. In 2018, about 160,000 children (0-9) lived with HIV, with 89,000 under five infected primarily during pregnancy and 76,000 while breastfeeding in Nigeria [6]. Breastfeeding accounts for between 25% and 44% of vertical transmission of HIV [7]. Globally, HIV-related maternal mortality ranges from 7% to 21%, with sub-Saharan regions having higher rates [8]. Additionally, HIV/AIDS has been linked to roughly 24% of maternal deaths in the sub-Saharan region [8].

Exclusive breastfeeding is the giving of breast milk without extra nourishment [9]. The dilemma of infant feeding decisions for mothers with HIV arises because the majority of HIV-1 transmission through breast milk occurs during the first four months of life, when breastfeeding has the greatest benefits and replacement feeding carries the highest risk of increasing infectious disease morbidity [1,10]. Breastfeeding promotion is a key component of child health initiatives worldwide due to its prominent and evident health benefits [11,12]. It is widely practiced in Nigeria because it is socially, culturally, and historically suitable [13,14]. However, since CTvertical transmission is the main way that young children contract HIV, this has presented a public health dilemma in the context of the HIV epidemic [1,7,15]. Decisions about the best mode of infant feeding in developing countries can be difficult because of social, economic and realistic constraints [16,17]. The relative risks of replacement feeding morbidity and mortality differ according to the environment, the mother's and her family´s circumstances, including her education and economic status [18-20]. Four 'pillars' were at the heart of the World Health Organization's (WHO) and the Joint United Nations Program on HIV/AIDS' (UNAIDS) strategic response to preventing HIV infection in infants: primary prevention of disease in women in general, prevention of HIV-infected women's transmission to their infants, treatment and care provision, and support for HIV-infected women and their families [10,21]. Thus, the option most likely to be chosen by HIV-infected women who do not wish to risk breastfeeding their infants is replacement feeding with formula or other foods [2,7,22].

However, when replacement feeding is acceptable, practical, affordable, sustainable, and safe, the WHO advised HIV-positive women to refrain from breastfeeding (AFASS) [23]. Replacing breastfeeding with baby formula is still a persisting challenge. Alvarenga *et al*. highlighted challenging maternal experiences of childhood formula practice, including psychological discomfort, the punitive feeling of inability to breastfeed, humiliation, and physical and mental suffering [24]. According to studies, breastfeeding alternatives that employ infant formula must be readily available, well-liked, economically feasible, and safe for children's normal growth and development [25-27]. This means that the formula must be manufactured in sanitary settings with an appropriate supply of clean water [28]. Even though Antiretroviral Therapy (ART) is advised for all HIV-positive mothers, and notably, EBF, to prevent vertical transmission of HIV, adherence to the recommended infant feeding guidelines (IFG) among women living with HIV has been inconsistent with varying factors [29]. These factors include the women´s knowledge of the guidelines, the spouse´s influence/perception of the recommended infant feeding practice, the family´s perception of the feeding guidelines and other sociocultural or religious influences [10,18,20,30]. Mother-to-child transmission rates have dramatically decreased with the recent suggestion that all HIV-positive childbearing women receive ART [9]. Antiretroviral Therapy (ART) decreases the viral load and thus decreases the risk of vertical transmission by exposing the maternal fluid to the infant's body [22].The prevention of vertical transmission of HIV is greatly aided by infant feeding programs. In high-income nations, formula feeding is advised in addition to breastfeed prevention as a means of preventing HIV transmission after birth.

However, formula use may be dangerous in low- and middle-income nations because of situations that make formula feeding unacceptable, impractical, expensive, sustainable, or safe, which increases the death rate for children under five [22,31]. Therefore, EBF concurrently with ARTs benefits reduces transmission and child mortality [18]. The most recent WHO HIV and infant feeding guidelines thus endorse EBF in areas where resources are scarce, with an optimal balance between preventing malnutrition and minimizing the vertical risk of HIV transmission [9]. Even when ART is unavailable, EBF is still encouraged, as evidence suggests that the majority of HIV-exposed children who are exclusively breastfed do not contract the virus [22,24,32]. Mixed feeding is thought to affect the integrity of gastrointestinal mucus and potentially enhance vertical transmission of HIV during breastfeeding [22]. In contrast, EBF decreases the risk of vertical transmission of HIV by 4 to 10-fold compared to mixed feeding [29]. The latest recommendation is to breastfeed until 12 months of age and then consider stopping breastfeeding for longer [9]. Due to testing available through initiatives for the prevention of vertical transmission of HIV, the majority of women in sub-Saharan Africa receive their HIV status during pregnancy. Since its inception in Nigeria in 2001, the program has expanded its reach and breadth multiple times, mostly in secondary and tertiary healthcare facilities. In spite of all of this, enrollment and involvement have been quite low [21,33]. This study examined the awareness, knowledge, and adherence to EBF while on antiretroviral treatment with viral load suppressed and the associated factors of EBF among mothers living with HIV in Port Harcourt, Nigeria.

## Methods

**Study design and setting:** the analysis in this paper was drawn from a multi-country community-based participatory research conducted between March and August 2022 among a cross-section of Black mothers living with HIV in Port Harcourt, Nigeria, Miami, Florida, USA and Ottawa, Canada. This paper focused on a cross-section of mothers in Port Harcourt, Nigeria [34]. Port Harcourt is the capital of Rivers state, which is situated in southern Nigeria. It is located 41 miles (66 km) upstream of the Gulf of Guinea along the Bonny River, which is a distributary of the Niger River in the east. It was established in 1912 in a previous home region to the Ijaw and Ikwere people [33]. It was selected because it is one of the states in Nigeria with a high percentage of persons living with HIV/AIDS [33].

**Study participant:** the research population used in this analysis consists of mothers who had one or more babies within the past ten years of living with HIV, and residing in Port Harcourt, Nigeria. Hence, inclusion criteria for this analysis included self-identification as a mother who had a baby for the past ten years of living with HIV, residing in Port Harcourt, Nigeria.

**Sample size estimation:** Taro Yamane formula calculated the sample size to be 340 participants at a confidence level of 95% and a 5% margin of error for every question [35]. The formula states that: Where: n=Sample size; N=Population size; e=marginal error


$$n=\frac{N}{1+N{\left(e\right)}^{2}}$$

To account for non-response and attrition, lower sample variability, the sample size was increased by 15%. A convenience sampling technique was employed to compile the necessary number of research participants. This non-probability sampling method chooses individuals based on their accessibility [36]. After data collection, 400 respondents were selected for data analysis, and only questionnaires with all the parts filled out by respondents were utilized.

**Study instrument:** the researcher created the questionnaire to answer the study questions because none already existed. The completed questionnaire was developed and modified accordingly to the prevention of vertical transmission of HIV guideline and validated by two public health specialists on HIV/AIDS, an epidemiologist, and a registered psychometrician. Twenty persons who were chosen via the face-to-face survey method also participated in a pilot test of the survey instruments; however, the results were not included in the study. The Cronbach's alpha was used to gauge its dependability. Its credibility was confirmed by the result, which was 0.85. The survey tools were divided into two parts: respondents' sociodemographic characteristics, such as respondents' personal information (Age, sex, marital status, education, employment, median household income, and ethnicity) and the second part, respondents were assessed on the awareness, knowledge, and adherence of the national guidelines for infant breastfeeding practices. To assess knowledge, participants were asked to state, based on a 1-item question, which of the following is the correct National policy or guideline about how to feed your child in the first year of giving birth when you are HIV positive? Mothers who chose the option, “Exclusive breastfeeding only” were regarded as knowledgeable about the national guidelines on EBF when living with HIV; in line with WHO recommendation to promote and support breastfeeding and antiretroviral interventions [9].

**Sampling procedure:** participants were identified using venue-based sampling, which involved engaging with community HIV support groups, community health centers, and outreach programs such as health fairs. Additionally, recruitment was supported through posted flyers and word-of-mouth referrals, leveraging networks within HIV care services. Healthcare workers and community outreach personnel played a crucial role in identifying potential participants who met the study's inclusion criteria. This non-probability sampling approach focuses on accessible populations and does not allow for random selection; therefore, the findings are specific to the study population and may not be directly extrapolated to all mothers living with HIV in Port Harcourt. Eligible participants were personally invited to take part in the study after being informed about its objectives, potential risks, and benefits. Invitations were extended during routine visits to health centers, through direct interactions at community support groups, and via outreach activities. Those who agreed to participate provided voluntary informed consent before completing the survey. For inclusivity, verbal explanations of the questionnaire were offered to participants with varying literacy levels to ensure accurate responses. Participants were recruited from community-based healthcare facilities, HIV treatment and prevention centers, and organized community gatherings focused on HIV awareness and support. These locations were chosen to ensure accessibility and familiarity for the target population, encouraging participation from mothers who had given birth while living with HIV. Only those who signed the informed consent form were included in the study. The questionnaire, risks and benefits of the survey were explained to participants who provided voluntary informed consent. It took 10 to 15 minutes for each respondent to complete the questionnaire. We verbally explained the questionnaire contents to some participants to get the correct responses, and for equal representation of mothers with various literacy levels. Those who did not sign the informed consent were excluded from this study.

**Data analysis:** a Microsoft Excel spreadsheet was used for data entry, editing, sorting, and coding. All the data collected were analyzed with the Statistical Package for Social Sciences, SPSS v25 (IBM, USA). The demographic distribution and adherence levels were presented with descriptive statistics (mean, frequency, and percentage). Chi-square statistic was used to test the association of selected factors with adherence to EBF among the mothers. Bivariate logistic regression analysis was also done to assess the odds of EBF. All research was done at a 95% confidence interval, and a p-value less than 0.05 was considered statistically significant.

**Ethical consideration:** the study received ethical approval from the University of Port Harcourt's Nigerian health research ethics council. Authorization Number: UPH/CEREMAD/REC/04). The participants consented to anonymize their replies. Only those who agreed to participate were allowed to fill up the form. Research ethics approvals were obtained from other study locations from which other datasets for the broader study were collected. These include the Ethics Boards of the University of Ottawa Canada (certificate #H08-16-27), Carleton University Ottawa (certificate #106300), and the Florida International University Miami-Florida, USA (certificate #105160).

## Results

**Sociodemographic characteristics of the mothers:** the majority of the respondents were within the age range 30-39 years, 264 (66.0%) with a mean age of 34.6±5.6, the youngest being 18 years old and the oldest 49 years old, married, 340 (85.6%), Christian, 390 (98.2%), have a secondary level of education, 239 (59.75%) and are employed or self-employed full-time, 272 (68.01%) with an average income of $1068.8 ±2321.0, the lowest income being $189.9 and the highest income $26,528. The median number of persons in a household was 4, ranging from 1 to 10. The median number of children born after HIV+ diagnosis was 2 (1-5), with an average number of years of HIV diagnosis of 6.3 ±3.5 years (Table 1).

**Table 1:** demographic characteristics of study participants recruited from community HIV support groups, community health centers, and outreach programs in Port Harcourt, Nigeria, between March and August 2022 (N=400)

Variable	Frequency	Percent (%)
**Age**		
≤19	4	1.00
20-29	54	13.50
30-39	264	66.00
40-49	78	19.50
**Age M (SD)**	34.67 ± 5.65	
Marital status (n=399)*		
Single	31	7.77
Married	340	85.21
Separated/divorced/widowed	26	6.52
Others	2	0.50
Religion (n=397)*		
Christian	390	98.2
Muslim	2	0.5
Mixed religion	5	1.3
**Level of education (n=400)**		
Primary education	41	10.25
Secondary education	239	59.75
Vocational or technical education	13	3.25
College or some university education	48	12.00
Completed degree programme	51	12.75
Completed masters	8	2.0
Employment status (n=365)*		
Not working, actively looking for work	38	9.5
Not working due to disabilities	1	.3
Working as a volunteer	4	1.0
Employed or self-employed part-time	50	12.5
Employed or self-employed full time	272	68.0
**Income in last one year (US$), M(SD)**	1068.8±2321.0	
Number of years since diagnosed HIV+, M (SD)	6.31 ± 3.54	
Number of persons in a household, MED (Min, Max)	4 (1-10)	
Number of children born after HIV+ diagnosis, MED (Min,Max)	2 (1-5)	

**M:** Mean, **SD**: standard deviation; **MED**: median; **Min**: minimum; **Max**: maximum; *Numbers reported do not add up to 400 because of participants who respond “I chose not to answer”

**Treatment, disclosure and knowledge of modes of transmission of HIV:** most mothers reported being on ARV treatment 397 (99.5%) and that they had talked to their spouses about their HIV status. About 68% of the respondents have spoken to their families about their HIV status. Precisely, 352 (89.3%) agree that vertical transmission of HIV can occur during pregnancy.

**Awareness and knowledge of the national guideline on infant feeding for mothers living with HIV:** most mothers were aware of the national guideline on feeding their child in the first year of giving birth when HIV positive, 362 (90.7%), and most got this information through the community/social worker, 304 (84.7%). Also, most mothers have correct knowledge of the national guideline for infant feeding for mothers living with HIV, 256 (71.1%).

**Adherence to the National policy or guideline for infant feeding for women living with HIV:** the distribution of the mothers' views on the national guideline on infant feeding practices for women living with HIV. A little over half, 214 (65.0%) of the mothers adhered to the EBF guideline, while 114 (35.0%) were non-adherent.

**Social supports received by the mothers living with HIV towards exclusive breastfeeding practices:** most of the respondents said the household support they receive towards making decisions about feeding their baby comes from their spouse/partner/baby´s father, 304 (76.8%), followed by support from mother/mother-in-law, 18 (4.6%), and the least from other relatives, 8 (2.0%). A little over half (68.1%) received support for EBF from their spouse/partner/baby´s father. However, they felt that the opinions of the spouse/partner/baby´s father were either unimportant (43.4%) or very unimportant (41.4%). A little below half (48.5%) received support towards EBF practices from their close relatives and cared less about their close relatives' opinions (45.5%). Healthcare provider support towards EBF practices were high (87.3%), and a higher percentage of the mothers had cultural beliefs about infant feeding practices (93.4%) (Table 2).

**Table 2:** characteristics of support for exclusive breastfeeding guidelines among mothers living with HIV recruited from HIV support groups, health centers, and outreach programs in Port Harcourt, Nigeria, between March and August 2022 (N=400)

Variable	Frequency	Percent
Receives household support in making decisions about feeding your baby from the following (n=396)*		
No one	66	16.7
My spouse/partner/baby’s father	304	76.8
My mother, mother-in-law	18	4.6
Other close relatives	8	2.0
Spouse/partner/baby’s father support towards EBF (n=266)*		
Yes	181	68.1
No	85	31.9
**How important is spouse/partner/baby’s opinion (n=263)**		
Important	10	3.8
Unsure	30	11.4
Unimportant	114	43.4
Very Unimportant	109	41.4
Close relatives (e.g., mother, mother-in-law, grandmother) support towards EBF (n=235)*		
Yes	114	48.5
No	121	51.5
**How important is close relatives opinion (n=235)**		
I do care	60	25.5
Unsure	36	15.3
I don’t care	107	45.5
I don’t care at all	32	13.6
I choose not to answer		
Healthcare provider support towards EBF (n=308)*		
Yes	269	87.3
No	39	12.7
**How important is the healthcare provider opinion (n=308)**		
I do care	3	1.0
I don’t care	95	30.8
I don’t care at all	210	68.2
There exist cultural beliefs/traditions about the methods of feeding baby (n=393)*		
Yes	367	93.4
No	26	6.6

* Numbers reported do not add up to 400 because of participants who respond, “I chose not to answer”

**Exclusive breastfeeding and associated factors among the mothers:** adherence to EBF practices varied across sociodemographic and other associated variables. For instance, 188 (68.2%) of the mothers that were employed adhered to EBF practices, compared to the 25 (49.0%) that were not employed (p=0.009). Different forms of support towards EBF improved adherence with varying proportions, compared to those not receiving support; household support (69.0% vs. 48.2%, p=0.003), spouse/partner support (92.2% vs. 15.3%, p=0.001), close relative support (81.4% vs. 55.0%), and Healthcare provider support (77.2% vs. 8.16%, p=0.001). Those saying cultural beliefs/traditions about the methods of feeding baby exist were above half for adherence compared to non-adherence (57.89% vs. 65.13%). However, findings were not statistically significant (p=0.522), but those with the correct knowledge of the guideline statistically significantly adhere more (73.75% vs. 30.77%, p=0.001). No statistically significant association was observed between age, marital status, level of education and adherence to EBF. Results of bivariate logistic regression analysis reveals that, being employed (OR: 2.22, 95% CI: 1.2-4.1; p=0.01), receiving support from the household (OR: 2.39, 95% CI: 1.3-4.3; p=0.003), receiving support from father´s/spouse (OR: 65.7, 95% CI: 29.4-146.7; p=0.001), receiving support from close relatives (OR: 3.6, 95% CI: 1.9-6.5; p=0.001), receiving support from the Healthcare (OR: 38.2, 95% CI: 13.2-110.3; p=0.001) and correct knowledge of the guideline (OR: 6.3, 95% CI: 3.0-13.2; p=0.001) predicted EBF practices at statistically significant levels (Table 3). After testing for confounders, using the multivariate logistic regression, only support from father/spouse (OR: 23.24, 95% CI: 7.7-70.2; p=0.001), and healthcare (OR: 47.6, 95% CI: 4.7-484.3; p=0.01) were strong predictors of adherence to EBF practices.

**Table 3:** factors associated with adherence to EBF guidelines among mothers living with HIV recruited from HIV support groups, health centers, and outreach programs in Port Harcourt, Nigeria between March and August 2022 (N=400)

Variables	Adherent	Non-adherent	χ^2^ (p-value)	Bivariate cOR [95% CI] p-value)	Multivariate aOR [95% CI] (p-value)
	**N=214**	**N=114**			
**Employment status**					
Employed or self-employed	188 (68.12)	88 (31.88)	6.91 (0.009*)	2.22 (1.21-4.07) (0.001*)	2.58 (0.75-8.93) (0.135)
Not working	25 (49.02)	26 (50.98)			
**Household support towards EBF**					
Yes	187 (69.0)	84 (31.0)	8.87 (0.003*)	2.39 (1.33-4.29) (0.003*)	3.65 (0.79-6.83) (0.096)
No	27 (48.21)	29 (51.79)			
**Spouse/partner support towards EBF**					
Yes	166 (92.22)	14 (7.78)	155.87 (0.001)*	65.67 (29.39-146.740)	23.24 (7.70-70.17)
No	13 (15.29)	72 (84.71)		146.74) (0.001*)	(0.001*)
**Close relatives support towards EBF**					
Yes	92 (81.42)	21 (18.58)	18.61 (0.001*)	3.58 (1.98-6.49) (0.001*)	1.28 (0.42-3.93) (0.662)
No	66 (55.0)	54 (45.0)			
**Healthcare provider support towards EBF**					
Yes	207 (77.24)	61 (22.76)	88.80 (0.001*)	38.18 (13.20-10.38) (0.001*)	47.69 (4.69-48.34) (0.001*)
No	4 (8.16)	45 (91.84)			
**Correct knowledge of the guideline**					
Yes	191 (73.75)	68 (26.25)	28.82 (0.001)*	6.32	4.53 (0.91-22.47)
No	12 (30.77)	27 (69.23)		(3.03-13.17) (0.001*)	(0.069)

* Statistically significant (p< 0.05), **χ^2^**=Chi-Square;), **cOR**: crude odds ratio; **aOR**: adjusted odds ratio CI: confidence interval; EBF: exclusive breastfeeding

## Discussion

“The WHO EBF guidelines for mothers living with HIV is aimed at prevention of vertical transmission while providing the infant with a nutritionally adequate and safe diet (breast milk). This study shows that most mothers living with HIV in Port Harcourt were aware, knowledgeable, and adhering to the EBF guidelines. Sociodemographic, family support system, spouse support, HIV treatment, Hub support system, and counselling were associated with adherence to the EBF guideline.

**Awareness and correct Knowledge of exclusive breastfeeding guidelines by mothers living with HIV:** the study shows that most mothers were aware (362 =90.7%) and knowledgeable about the EBF guideline. This finding corroborates similar studies [1,19,20,29,37,38]. These findings are similar to the reports of other studies in sub-Saharan Africa, which indicate that about 50 - 90% of women living with HIV have comprehensive knowledge of vertical transmission of HIV [32,39-41]. The relatively high proportion of respondents with good knowledge of vertical transmission of HIV has been attributed to the different awareness campaign programs and continuous HIV/AIDS education ongoing in various parts of sub-Saharan Africa [10,42,43].

**Adherence to the exclusive breastfeeding guidelines among mothers living with HIV:** the study showed that a little above half (67%) of the respondents were currently abiding by the recommended infant feeding practice, and about a third (33%) were not adherent to the IFGs, which is similar to the report of Mohammed *et al*. [33] where mothers in Abuja, Nigeria, followed recommended feeding practices for their infants at a rate of 46%. The current study's findings are also similar to a survey in southeast Nigeria which showed that 68% of the women adhered to the guidelines [20]. A related study by Belay and Wubneh reported a 63% adherence to IFGs by nursing mothers living with HIV in Ethiopia [2]. Compared to these other findings, the adherence to IFGs among the women in Port Harcourt seems equally proportional. The region's awareness of HIV among nursing mothers living with HIV is relatively high. This is mainly attributed to the HIV testing done for mothers attending pre-natal care at different health centers. During prenatal care, these women are given adequate information on preventing mother-child transmission [24,32].

**Employment status and adherence to exclusive breastfeeding practices by mothers living with HIV:** the mothers who were in paid employment or self-employed were 2.22 times more likely to adhere to EBF guidelines. This result is corroborated by data from the 2008 Nigerian demographics and health census [44], which found that women working in private or public sectors where breastfeeding is encouraged or who are self-employed were more likely to breastfeed their children appropriately. The study supports findings that being of higher socio-economic status, which is linked to being employed, promotes adherence to IFGs. The study advocated that the international code of marketing of breastmilk substitutes in Nigeria be strengthened, as well as other national policies that benefit nursing mothers in the workplace, such as the provision of crèches, paid parental leave, and prolonged paid maternal leave. However, in contrast to the present study findings, reports of Nyoni *et al*. showed that most women who adhered to the recommended IFGs are unemployed [22]. It is understood that those with full-time employment in low-middle-income countries have reported mixed feeding practices to accommodate their job schedules due to the relatively lesser number of maternity leave days they have compared to women in high-income countries [7,18]. The average maternity leave of employed women in low-to-middle countries varies between 6 - 12 weeks which is in stark contrast to the average maternity leave in developed countries, which is at least 12 weeks [1,29]. Also, public breastfeeding or breastfeeding at the workplace in sub-Saharan Africa is considered low due to the inadequate policy to support it and poor perceptions of public or workplace breastfeeding practice [1,7,45].

**Knowledge of exclusive breastfeeding guidelines and adherence among mothers living with HIV:**in the present study, adherence was positively and substantially correlated with knowing the right newborn feeding recommendations among HIV moms. In other words, among the mothers, knowledge of the recommendations was associated with a 6.32 chance of following the guideline. This is similar to the reports of previous studies, which showed that adequate knowledge of the recommended feeding guidelines was essential to the appropriate practice among mothers living with HIV in sub-Saharan Africa [7,8,10,18]. In a study by Itiola *et al*. knowledge of feeding guidelines has also been shown to be significantly associated with adherence to feeding guidelines (AOR: 2.4, 95% CI: 1.1 - 5.5, p = 0.032) [1]. In recent years, donor intervention and increased participation of local health authorities have shown some increase in awareness of HIV transmission and treatment in Africa. The recent increase in HIV/AIDS campaigns has given mothers living with HIV in Africa increased hope and access to treatment to improve their standards of living [24,10,22]. Family and healthcare providers' opinions as factors associated with exclusive breastfeeding while on Anti-retroviral treatment among mothers living with HIV. In the present study, the majority of the respondents chose to inform their spouse of their current HIV status, than their other family members, as observed in the present study, which supports the findings from similar studies [42,43,46,47]. Mother´s agreeing with the father´s/spouse´s support concerning the guidelines was positively and significantly associated with adherence to EBF while on ART.

Consequently, this led to an increased likelihood of adherence. It has been reported that the spouse/father of the child´s support goes a long way in influencing the self-esteem of women living with HIV in high-income and low-middle-income countries worldwide [1,29]. In other words, spousal support is a vital factor associated with successful adherence to treatment and IFGs among these women. A study by Umeobieri *et al*. [20] reported an increased likelihood of infant feeding adherence among women living with HIV in South-East Nigeria (OR; 6.5; 3.2 - 12.9). Also, a study by Mugwanya *et al*. [10] reported that spousal and family support significantly influenced breastfeeding practices among women living with HIV in Kenya. Our study also showed that mothers that agree with the health provider´s support towards the guideline were 33.39 times more likely to adhere to the IFG (p=0.002). Studies by West *et al*. [7], Mugwanya *et al*. [10], and Mohammed *et al*. [33] showed that health workers' opinions have been significant in the practice of recommended infant feeding policies among women living with HIV in Africa. This study has revealed that spousal support, family support, and the health workers' opinions are essential in influencing infant feeding practices among women living with HIV. The proportion of adherent respondents was also significantly higher than the non-adherent respondents when the family members and the health provider´s opinions agreed with the guidelines. This also was associated with an increased likelihood of adherence among the respondents. Serovich *et al*. [43] reported that a significant proportion of women (~40- 60%) living with HIV in Africa tend not to inform extended family members of their status. The fear of unintentional disclosure and the resultant HIV stigma makes many women not adhere to antiretroviral treatment and IFGs [46]. Non-adherence to guidelines has serious negative implications for infant feeding patterns among women living with HIV [20,22,45], including vertical transmission, child malnutrition, and mortality.

**Limitations of the study:** the study's cross-sectional design limits the ability to establish causal relationships between adherence to exclusive breastfeeding (EBF) and the associated factors identified. Additionally, the use of venue-based, non-random sampling means that findings are specific to the study population and may not be generalizable to all mothers living with HIV in Port Harcourt. The results should be interpreted with caution, as they reflect the experiences of participants recruited from specific community health centers, HIV support groups, and outreach programs. Furthermore, recall bias may have affected the accuracy of participants' self-reported infant feeding practices. Some mothers may have unintentionally misreported or selectively recalled their feeding behaviors due to social desirability bias or difficulties in remembering past actions. Future studies employing longitudinal designs with randomized sampling may enhance generalizability and provide a clearer understanding of the factors influencing EBF adherence in broader populations.

## Conclusion

Most women living with HIV adhered to IFGs and were knowledgeable of the risk of vertical transmission of HIV through breast milk. Awareness of the guidelines and the opinions of spouses, other family members, and healthcare professionals were important factors in adherence to IFGs among mothers living with HIV. We recommend a strengthened critical health literacy intervention to promote the knowledge, understanding application of the infant feeding guidelines among mothers living with HIV, their families, health service providers, and their communities. Including the family members, health providers, and communities in the guideline´s enlightenment programs will increase social support and medical support received by mothers living with HIV and improve their adherence to treatment and IFGs. While the findings provide valuable insights, they are specific to the study population and may not fully represent all mothers living with HIV in Port Harcourt.

### What is known about this topic

- Studies have shown that exclusive breastfeeding guidelines prevent mother-to-child HIV transmission;
- According to experts, understanding prevention of vertical transmission of HIV will aid in minimizing its prevalence rates among children worldwide;
- Previous research has found that women who adhere entirely to antiretroviral treatment have low CD4 count, reducing the chances of infecting their children.

### What this study adds

- This study reveals that among the study population of mothers living with HIV in Port Harcourt, Nigeria, recruited from HIV support groups, health centers, and outreach programs, most were aware of the exclusive breastfeeding guidelines; however, adherence to these guidelines was observed in just a little over half of the participants;
- According to the study findings, employed/self-employed mothers living with HIV in the study population were more likely to adhere to exclusive breastfeeding guidelines compared to those without employment; further research could explore the underlying reasons for this association;
- Adherence to exclusive breastfeeding guidelines among study participants was strongly associated with support from spouses, relatives, and healthcare providers; this highlights the critical role of social and medical support systems in promoting adherence among mothers living with HIV.

## References

[1] Itiola AJ, Goga AE, Ramokolo V (2019). Trends and predictors of mother-to-child transmission of HIV in an era of protocol changes: findings from two large health facilities in North East Nigeria. PLoS One.

[2] Belay GM, Wubneh CA (2019). Infant Feeding Practices of HIV Positive Mothers and Its Association with Counseling and HIV Disclosure Status in Ethiopia: A Systematic Review and Meta-Analysis. AIDS Res Treat.

[3] Adeyemo BO, Gayawan E, Olusile AO, Komolafe IO (2014). Prevalence of HIV infection among pregnant women presenting to two hospitals in Ogun state, Nigeria. HIV & AIDS Review.

[4] Joint United Nations Programme on HIV/AIDS (2021). New survey results indicate that Nigeria has an HIV prevalence of 1.4%. UNAIDS.

[5] United Nations International Children's Emergency Fund (2021). HIV/AIDS| UNICEF Zambia. UNICEF.

[6] United Nations International Children's Emergency Fund (2019). More than 47 Nigerian children and adolescents die every day from AIDS-related causes. UNICEF.

[7] West NS, Schwartz SR, Yende N, Schwartz SJ, Parmley L, Gadarowski MB (2019). Infant feeding by South African mothers living with HIV: implications for future training of health care workers and the need for consistent counseling 11 Medical and Health Sciences 1117 Public Health and Health Services. Int Breastfeed J.

[8] Moseholm E, Weis N (2020). Women living with HIV in high-income settings and breastfeeding. J Intern Med.

[9] World Health Organization (2010). Guidelines on HIV and infant feeding. WHO.

[10] Mugwanya KK, Hendrix CW, Mugo NR, Marzinke M, Katabira ET, Ngure K (2016). Pre-exposure prophylaxis use by breastfeeding HIV-uninfected women: a prospective short-term study of antiretroviral excretion in breast milk and infant absorption. PLoS Med.

[11] Pérez-Escamilla R, Martinez JL, Segura-Pérez S (2016). Impact of the Baby-friendly Hospital Initiative on breastfeeding and child health outcomes: a systematic review. Matern Child Nutr.

[12] Hitayezu J, Ntirushwa D, Ntiyamira JC, Kayitesi J, Murungi RM, Olufolabi AJ A cross-sectional study to evaluate adherence to the ten steps to successful breastfeeding at a Referral Hospital in Rwanda.

[13] Qureshi AM, Oche OM, Sadiq UA, Kabiru S (2011). Using community volunteers to promote exclusive breastfeeding in Sokoto State, Nigeria. Pan Afr Med J.

[14] Ajuba M Nwala E (2015). Scaling up exclusive breastfeeding among mothers in Enugu East local government area Nigeria: a health intervention study. Int J Sci Res.

[15] Okonko IO, Osadebe AU, Onianwa O, Okereke S (2019). Prevalence of HIV in a Cohort of Pregnant Women Attending a Tertiary Hospital in Ibadan, Nigeria. Immunology and Infectious Diseases.

[16] Agunbiade OM, Ogunleye OV (2012). Constraints to exclusive breastfeeding practice among breastfeeding mothers in Southwest Nigeria: Implications for scaling up. Int Breastfeed J.

[17] Ahishakiye J, Bouwman L, Brouwer ID, Matsiko E, Armar-Klemesu M, Koelen M (2019). Challenges and responses to infant and young child feeding in rural Rwanda: a qualitative study. J Health Popul Nutr.

[18] Acheampong AK, Naab F, Kwashie A (2018). The voices that influence hiv-positive mothers´ breastfeeding practices in an urban, ghanaian society. J Hum Lact.

[19] Dutta N, De R, Pain S, Modak D, Guha SK (2014). A sequential approach to identify the offending medication in HIV sero-positive patients having hypersensitivity to fixed dose combination of HAART. HIV and AIDS Review.

[20] Umeobieri AK, Mbachu C, Uzochukwu BSC, Elias A, Omotowo B, Agunwa C (2018). Perception and practice of breastfeeding among HIV positive mothers receiving care for prevention of mother to child transmission in South-East, Nigeria. Int Breastfeed J.

[21] Arrivillaga M, Arroyave BE, Salcedo JP (2014). Resilience processes in women leading community based organizations providing HIV prevention services. HIV & AIDS Review.

[22] Nyoni S, Sweet L, Clark J, Ward P (2019). A realist review of infant feeding counselling to increase exclusive breastfeeding by HIV-positive women in sub Saharan-Africa: What works for whom and in what contexts. BMC Public Health.

[23] United Nations International Children's Emergency Fund. Joint United Nations Programme on HIV/AIDS (UNAIDS), World Health Organization HIV and infant feeding Guidelines for decision-makers.

[24] Alvarenga WA, Nascimento LC, Leal CL, Fabbro MRC, Bussadori JCC, Melo SSES (2019). Mothers living with HIV: replacing breastfeeding by infant formula. Rev Bras Enferm.

[25] Green M, Pries AM, Hadihardjono DN, Izwardy D, Zehner E, Moran VH (2021). Breastfeeding and breastmilk substitute use and feeding motivations among mothers in Bandung City, Indonesia. Matern Child Nutr.

[26] Barennes H, Slesak G, Goyet S, Aaron P, Srour LM (2016). Enforcing the International Code of Marketing of Breast-milk Substitutes for Better Promotion of Exclusive Breastfeeding. J Hum Lact.

[27] Champeny M, Pries AM, Hou K, Adhikary I, Zehner E, Huffman SL (2019). Predictors of breast milk substitute feeding among newborns in delivery facilities in urban Cambodia and Nepal. Matern Child Nutr.

[28] Gribble KD, Hausman BL (2012). Milk sharing and formula feeding: Infant feeding risks in comparative perspective?. Australas Med J.

[29] Greene S, Ion A, Elston D, Kwaramba G, Smith S, Carvalhal A (2015). “Why Aren´t You Breastfeeding?”: how mothers living with HIV talk about infant feeding in a “Breast Is best” World. Health Care Women Int.

[30] Etowa J, Hannan J, Etowa EB, Babatunde S, Phillips JC (2021). Determinants of infant feeding practices among Black mothers living with HIV: a multinomial logistic regression analysis. BMC Public Health.

[31] Kafulafula UK, Hutchinson MK, Gennaro S, Guttmacher S (2014). Maternal and health care workers´ perceptions of the effects of exclusive breastfeeding by HIV positive mothers on maternal and infant health in Blantyre, Malawi. BMC Pregnancy Childbirth.

[32] Nkwabong E, Meboulou Nguel R, Kamgaing N, Keddi Jippe AS (2018). Knowledge, attitudes and practices of health personnel of maternities in the prevention of mother-to-child transmission of HIV in a sub-Saharan African region with high transmission rate: Some solutions proposed. BMC Pregnancy Childbirth.

[33] Mohammed A, Shehu AU, Aliyu A, Zoaka A (2010). Infant feeding options, practices and determinants of feeding practices among HIV seropositive mothers in Abuja, Nigeria. Niger Med J.

[34] Etowa J, Nare H, Kakuru DM, Etowa EB (2020). Psychosocial experiences of HIV-positive women of African descent in the cultural context of infant feeding: a three-country comparative analyses. Int J Environ Res Public Health.

[35] Joskow J (1965). Statistics, an introductory analysis. J Am Stat Assoc.

[36] Lavrakas P (2008). Encyclopedia of Survey Research Methods. Sage publications.

[37] Ogbe AC, Nwankwo CU, Agbele TO, Nwambo JC (2020). Socio-demographic determinants of exclusive breastfeeding practices among nursing mothers in Enugu State. Int J Stud Nurs.

[38] Skwara P, Bociąga-Jasik M, Kalinowska-Nowak A, Sobczyk-Krupiarz I, Garlicki A (2014). Adherence to single-tablet versus multiple-tablet regimens in the treatment of HIV infection: A questionnaire-based survey on patients satisfaction. HIV & AIDS Rev.

[39] Alemu YM, Habtewold TD, Alemu SM (2018). Mother´s knowledge on prevention of mother-to-child transmission of HIV, Ethiopia: a cross sectional study. PLoS One.

[40] Beyene GA, Dadi LS, Mogas SB (2018). Determinants of HIV infection among children born to mothers on prevention of mother to child transmission program of HIV in Addis Ababa, Ethiopia: A case control study. BMC Infect Dis.

[41] Luba TR, Feng Z, Gebremedhin SA, Erena AN, Nasser AM, Bishwajit G (2017). Knowledge about mother-to-child transmission of HIV, its prevention and associated factors among Ethiopian women. J Glob Health.

[42] Charurat M, Oyegunle M, Benjamin R, Habib A, Eze E, Ele P (2010). Patient retention and adherence to antiretrovirals in a large antiretroviral therapy program in Nigeria: a longitudinal analysis for risk factors. PLoS One.

[43] Serovich JM, Craft SM, Reed SJ (2012). Women’s HIV disclosure to family and friends. AIDS Patient Care STDS.

[44] Ogbo FA, Agho KE, Page A (2015). Determinants of suboptimal breastfeeding practices in Nigeria: Evidence from the 2008 demographic and health survey. BMC Public Health.

[45] Odiachi A, Erekaha S, Cornelius LJ, Isah C, Ramadhani HO, Rapoport L (2018). HIV status disclosure to male partners among rural Nigerian women along the prevention of mother-to-child transmission of HIV cascade: a mixed methods study. Reprod Health.

[46] Spangler SA, Onono M, Bukusi EA, Cohen CR, Turan JM (2014). HIV-positive status disclosure and use of essential PMTCT and maternal health services in rural Kenya. J Acquir Immune Defic Syndr.

